# Early Intervention services in the era of genomic medicine: setting a research agenda

**DOI:** 10.1038/s41390-024-03668-5

**Published:** 2024-10-22

**Authors:** Katherine E. MacDuffie, Betty Cohn, Paul Appelbaum, Kyle B. Brothers, Dan Doherty, Aaron J. Goldenberg, Elizabeth Reynolds, Hadley Stevens Smith, Anne Wheeler, Joon-Ho Yu

**Affiliations:** 1 Treuman Katz Center for Pediatric Bioethics and Palliative Care, Seattle Children’s Research Institute, Seattle, WA USA; 2 Department of Pediatrics, University of Washington School of Medicine, Seattle, WA USA; 3 Institute for Public Health Genetics, University of Washington, Seattle, WA USA; 4 Department of Psychiatry, Columbia University Irving Medical Center and NY State Psychiatric Institute, New York, NY USA; 5 Department of Pediatrics, University of Louisville, Louisville, KY USA; 6 Department of Bioethics, Case Western Reserve University School of Medicine, Cleveland, OH USA; 7 RTI International, Research Triangle Park, NC USA; 8 Precision Medicine Translational Research (PROMoTeR) Center, Department of Population Medicine, Harvard Medical School and Harvard Pilgrim Health Care Institute, Boston, MA USA; 9 Center for Bioethics, Harvard Medical School, Boston, MA USA

## Abstract

**Abstract:**

Newborn genomic sequencing (NBSeq) has the potential to substantially improve early detection of rare genetic conditions, allowing for pre-symptomatic treatment to optimize outcomes. Expanding conceptions of the clinical utility of NBSeq include earlier access to behavioral early intervention to support the acquisition of core motor, cognitive, communication, and adaptive skills during critical windows in early development. However, important questions remain about equitable access to early intervention programs for the growing number of infants identified with a genetic condition via NBSeq. We review the current NBSeq public health, clinical, and research landscape, and highlight ongoing international research efforts to collect population-level data on the utility of NBSeq for healthy newborns. We then explore the challenges facing a specific Early Intervention (EI) system—the US federally supported “Part C” system—for meeting the developmental needs of young children with genetic diagnoses, including structural limitations related to funding, variable eligibility criteria, and lack of collaboration with newborn screening programs. We conclude with a set of questions to guide future research at the intersection of NBSeq, newborn screening, and EI, which once answered, can steer future policy to ensure that EI service systems can optimally support the developmental needs of infants impacted by broader implementation of NBSeq.

**Impact:**

- Existing literature on the clinical benefits of genome sequencing in newborns tends to focus on earlier provision of medical interventions, with less attention to the ongoing developmental needs of very young children with genetic conditions.
- This review outlines the developmental needs of a growing number of children diagnosed with genetic conditions in infancy and describes the strengths and limitations of the United States Early Intervention system (IDEA Part C) for meeting those needs.

## Introduction

Rare genetic conditions, considered together, affect an estimated 300 million individuals worldwide and are the leading cause of child mortality and disability in high-income countries.^1–3^ Recognition of the substantial public health impact of such conditions led to the establishment of state-run newborn screening (NBS) programs in the USA in the 1960s. With the advent in recent decades of genome sequencing, newborn genomic sequencing (NBSeq)—used here to refer to genetic risk screening via sequencing of either the exome or the genome—has the potential to shift the landscape of early detection of rare genetic disorders. NBSeq would allow for screening of thousands of conditions at birth, including conditions not detectible through traditional NBS. Proponents of NBSeq emphasize the life-saving potential of identifying and treating newborns before they experience morbidity or mortality from a genetic condition.^4,5^ However, the utility of NBSeq as a universal screening method for healthy newborns has not yet been established.^5^ NBSeq raises a number of challenges, including the potential to expose families to uncertainty and other negative psychosocial impacts, further burden healthcare systems, and exacerbate health disparities.^6–9^

In this review, we focus on a specific challenge of NBSeq implementation: whether current service systems can equitably support the developmental needs of infants identified with genetic disorders via NBSeq. Developmental services like speech and language therapy, physical therapy, occupational therapy, and special education can support the acquisition of core skills for infants and toddlers with delayed development. Specifically, the US federally supported “Part C” system, often referred to as Early Intervention (EI), could provide additive benefits over and above medical treatments.^10–13^ However, due to geographic differences in EI systems, developmental supports may not be equally available to all infants with genetic diagnoses,^14,15^ and socioeconomically disadvantaged families may face particular challenges leveraging a genetic diagnosis to access necessary services.^16^

**Fig. 1:**
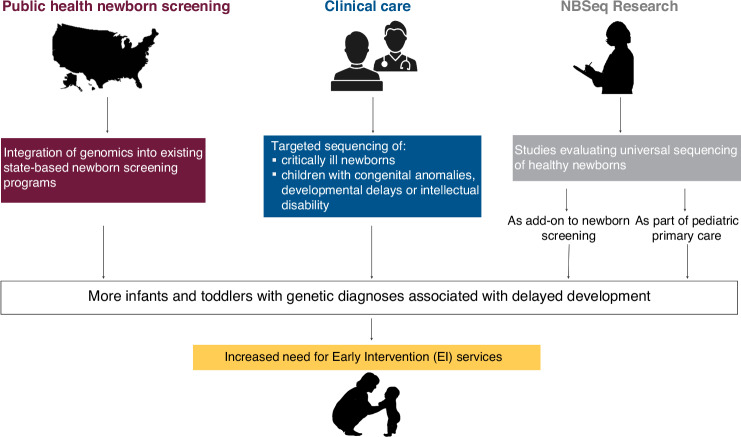
The public health, clinical, and research landscape for NBSeq.

**Fig. 2:**
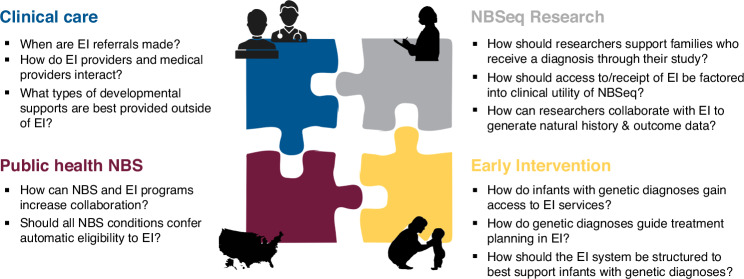
Questions for future research.

In four sections, we describe (1) the current public health, clinical, and research landscape for NBSeq (Fig. 1), (2) the developmental needs of children diagnosed with monogenic conditions, and (3) gaps in developmental services that are likely to impact the growing number of children with identified genetic conditions, with a particular focus on the US EI system. We conclude with (4) suggestions for future research to fill knowledge gaps and inform evaluations of the potential benefits and harms of implementing NBSeq at scale.

## Current public health, medical, and research landscape for NBSeq

### Public health

Since the 1960s, the USA has implemented NBS as a public health program intended to identify infants at birth who have serious medical conditions. Today, NBS programs include a hearing screen to detect hearing loss, a pulse oximetry screen to test for critical congenital heart defects, and most relevant for our purposes, a blood-spot test to detect genetic conditions. Newborns tend not to show symptoms of NBS conditions at birth; however, without timely treatment, these conditions could cause significant harm, or in some cases, death. Diagnosis of many conditions first flagged via NBS is considered a medical emergency.^17^

The blood-spot test is intended to detect genetic conditions. However, for the first 5 decades of state NBS programs, the technology employed did not test genes directly. Only since 2010 has DNA-based screening been included as a first-tier test in NBS programs (first used for the TREC assay to detect severe combined immunodeficiency or SCID).^18^ More recently a few states have begun to adopt genomic sequencing as part of tiered testing strategies.^19,20^ Incorporation of sequencing into existing NBS programs is a market opportunity for companies that are developing next-generation sequencing-based panels and workflows designed for NBS dried blood spots.^21^ The resulting large datasets could be leveraged or made available to researchers or industry partners working to develop therapeutics for rare disease.^22^ However, whether and when state-based NBS programs will begin to use genomic sequencing on a broader scale is debated, and integration would require substantial overhaul of current systems.^23,24^

### Clinical care

Clinical use of NBSeq as a diagnostic tool for infants already showing symptoms is quite different from its potential incorporation into universal, public-health NBS. The clinical setting in which diagnostic sequencing has been most broadly implemented thus far is in neonatal intensive care units (NICUs). When applied to critically ill newborns, diagnostic sequencing shows superior performance for detecting genetic conditions compared to conventional methods.^25–27^ The relatively high diagnostic rate and potential for immediate changes in medical management together make a particularly strong argument for the clinical utility of diagnostic sequencing in the NICU setting.

Beyond the NICU, genome sequencing is being increasingly implemented as a diagnostic test for young children with developmental delays.^7,28^ Evidence-based clinical guidelines from the American College of Medical Genetics and Genomics (ACMG) now strongly recommend genome sequencing as a first-tier test for all patients <1 year with one or more congenital anomalies and all patients <18 years with developmental delay or intellectual disability.^29^ However, not all insurers cover clinical sequencing, and without coverage out-of-pocket costs for families are often prohibitively high.^30^

### Research

A growing body of research studies have motivated clinical adoption of newborn sequencing for *diagnostic* purposes.^26^ However, the evidence base for NBSeq applied as a universal screening tool to healthy newborns is nascent. The first iteration of BabySeq, a pilot randomized clinical trial of genome sequencing that included a subsample of healthy newborns, found variants that conferred monogenic disease risk in 10/127 (8%) of healthy newborns who were sequenced.^31^ Follow-up analyses of medical actions taken 3–5 years after results disclosure determined that all genomic findings were moderately or highly actionable.^5^ However, the initial study was underpowered to assess clinical utility or cost-effectiveness, and like many genomic research studies, the sample lacked sociodemographic diversity. The second iteration of BabySeq^32^ addresses these issues by recruiting a larger sample with substantially modified recruitment protocols, developed with input from a community advisory board, and has reported initial rates of enrollment (17–25% of those approached enrolled) that are similar across racial/ethnic groups.^33^

To generate the larger and more diverse samples needed to assess the utility of universal screening via NBSeq, a number of international studies are currently evaluating the risks and benefits of employing universal sequencing, with parent consent, in population-based samples of healthy newborns (Table 1). Importantly, some of these studies are conducted in collaboration with national/state NBS programs, performing sequencing on the same dried blood spots already collected for NBS, with a goal to provide initial evidence that NBSeq could be feasibly integrated into NBS. Others are focused on the integration of NBSeq into pediatric primary care and require new blood sample collection. Participating families are required to provide informed consent for research in all of these studies, meaning that families who do not want to participate in research will not be represented in these large-scale efforts, potentially compromising their generalizability.

**Table 1:** Large-scale NBSeq research studies.

Study name	Location	Goal sample size	Number of genes or conditions screened	Uses existing NBS sample, or requires new collection	Launch date
Generation Study	England	100,000	250 conditions	New	2024
PERIGENOMED	France	20,000	150 conditions	–	2023
BabyScreen+	Australia	1000	500 conditions	Existing	2023
NewbornsinSA	Australia	40,000	600 conditions	Existing	2023
GUARDIAN	USA	100,000	450 conditions	Existing	2022
BeginNGS	USA, Greece	2000	500 conditions	Existing	2022
Baby Detect	Belgium	40,000	126 conditions, 363 genes	New	2022
Screen4Care	European Union	25,000	200 genes	–	2021
ScreenPlus	USA	100,000	14 conditions	Existing	2021
Early Check	USA	10,000	200 conditions	Existing	2018
BabyBeyond	Australia	106	222 genes	New	2016
NC Nexus	USA	400	–	New	2016
BabySeq	USA	1000	4300 genes	New	2015
NBSeq Team	USA	1200	78 genes	Existing	2013

Study details were derived, when available, from public websites or published materials.

Each research group has curated an independent list of genes and corresponding disorders to test for during the NBSeq process, ranging from a few hundred to over 1000 conditions. Of note, across 26 large-scale NBSeq programs, only 74 of a total of 1750 genes were included on >80% of gene lists.^34^ This variation is due to different condition inclusion criteria used by studies (e.g., focusing on gene-disease validity vs. treatability vs. age of condition onset). Across all studies, the top factors that predicted gene-list inclusion were a strong evidence base for the gene-disease pair, high disease penetrance, and availability of effective treatments.^34^ Efforts to develop consensus guidelines for gene-list inclusion, based on expert opinion,^35^ are ongoing.

Two studies, GUARDIAN and Early Check, adopted a model of allowing parents to decide the conditions for which their child should be screened using a tiered-consent process. These studies screen all enrolled children for a group of conditions designated as clinically actionable (Group 1) and ask parents to decide whether they would like their child to be screened for a group of additional conditions (Group 2: mostly neurodevelopmental conditions) for which treatments are still in development. Notably, GUARDIAN’s recruitment website specifically mentions EI in relation to Group 2 (non-treatable) conditions: “Early diagnosis of these conditions can support you and your child with EI services including physical therapy, occupational therapy, and speech therapy.”^36^ From publicly available information, it is unclear whether other studies factored EI/developmental supports into their definitions of actionable conditions to be included on gene-condition lists. In recognition of the need to standardize practices across studies, groups like the International Consortium on Newborn Sequencing (ICoNS) have formed with a goal to create common practices and policies for NBSeq efforts worldwide.

As the use of genome sequencing technology increases in national/state public health programs, clinical care, and research, it is likely that the number of infants and toddlers with genetic diagnoses confirmed by genome sequencing is increasing and will continue to do so. Many monogenetic conditions are associated with a significant risk for developmental delay and intellectual disability, which can be further exacerbated by clinical features of the condition (e.g., epilepsy).^37^ Regardless of whether sequencing was conducted because of evident delays or prior to their onset, young children diagnosed with genetic conditions represent a growing group of patients in need of developmental supports.

## Developmental needs of children with genetic disorders

The primary reason to employ genomic sequencing in the newborn period is to identify genetic risk so that appropriate treatment can be accessed as early as possible to reduce morbidity and mortality in early childhood. As described, NBS programs focus their efforts on genetic conditions that are treatable, consistent with the Recommended Uniform Screening Panel (RUSP)—a list of conditions recommended by the US Department of Health Human Services for inclusion on state screening panels.^38^ Conditions are added to the RUSP after a defined process weighing benefits (i.e., treatability), feasibility, and program readiness.^39^ Ultimately, decisions rely on the notion of medical “actionability”^40^—conditions that can be prevented, treated, or cured, via pharmacologic, dietary, surgical, or more recently, gene-based therapies. Developmental supports are not considered treatments or factored into RUSP inclusion decisions. However, as the utility of genome sequencing for identifying neurodevelopmental conditions has become evident, scholars have called for a broadening of the category of “treatment” to include interventions that target symptoms (e.g., anti-seizure medications) but are not curative of the underlying disorder, as well as developmental supports such as physical and occupational therapy, speech therapy, and special education.^6,41–44^

Most children with genetic conditions will require a combination of medical treatment and developmental support. An estimated 85% of RUSP conditions are likely to result in developmental delays and thus could benefit from supportive services.^14^ For example, spinal muscular atrophy (SMA)—a RUSP disorder affecting motor neurons—can lead to significant physical impairments due to muscle weakness and death in early childhood without treatment. Even with optimal medical treatment, children with SMA require developmental supports. Among children with SMA treated with nusinersen, those who also received physical therapy showed superior muscle strength, range of motion, and ability to perform daily tasks compared to those who received nusinersen without physical therapy.^11^

Providing developmental supports during early childhood, when brain plasticity and skill acquisition are at their lifetime peak, is critical. Emerging evidence, largely from preclinical animal models, suggests that providing interventions presymptomatically—prior to the onset of delays—can improve outcomes by taking advantage of critical windows in development.^45–49^ A randomized clinical trial for infants 9–15 months old showing very early signs of autism found that early behavioral intervention reduced autism diagnostic behaviors, resulting in fewer children meeting diagnostic criteria for autism at age 3.^48^ A preclinical mouse model of Rett syndrome—a progressive neurodevelopmental disorder characterized in humans by skill regression in the 2nd year of life—found that preemptive motor and memory training prior to the onset of symptoms improved task performance and delayed symptom onset; the same performance benefits were not observed if training started after symptoms had already appeared.^49^

The importance of supporting the acquisition of core developmental skills during the earliest years for infants with genetic diagnoses has led some scholars to suggest that a new system of specialty clinics be established to provide follow-up services for infants with genetic diagnoses.^37,50,51^ A more resource-efficient approach, we argue, would be to leverage existing service systems that already support the developmental needs of young children and equip those systems with the skills (and funding) needed to serve the growing number of children expected to receive genetic diagnoses during infancy as a result of newborn sequencing. In the next section, we explore one existing service system—the US EI system—and its potential for supporting the developmental needs of children with genetic diagnoses.

## Strengths and limitations of the US EI system

In the USA, a federally funded, state-administered system that supports the developmental needs of children under 3 years of age is authorized under the Individuals with Disabilities Education Improvement Act (IDEA) Part C. IDEA (originally the Education for All Handicapped Children Act) was established by Congress in 1975 with the intent to provide access to education for children ages 3–18 years with disabilities.^52^ The reauthorization in 1986 added “Part C” to support EI for infants and toddlers (age 0–2 years), responding to “an urgent and substantial need” to minimize the potential for developmental delay, reduce later special education costs, and build the capacities of families to meet their children’s needs.^53^ From 2022 to 2023, a cumulative total of 853,298 children ages 0–2 years were served in Part C EI across the USA and territories.^54^ Estimates based on concurrent census data suggest that Part C EI serves 3.7% of the total US population ages 0–2.

Services provided to children in Part C EI include physical therapy, occupational therapy, speech therapy, and early childhood education.^55^ Most services are provided in the home, but they can also take place in childcare or community settings, and are usually delivered weekly for a period of months to years.^56,57^ Part C legislation focuses explicitly on meeting the needs of minority and low-income children,^58^ with services provided at low or no cost to families, and no denial of services based upon inability to pay. In addition, the law requires that materials and procedures be provided in a family’s preferred language. Part C EI services are explicitly family-centered, and therapists employ a parent-coaching model to teach parents how to provide learning and skill-building opportunities as part of everyday routines with their child.^59^ Studies support the efficacy of Part C EI for improving outcomes for children with a wide range of delays and disabilities.^60–62^ Part C EI also benefits parents, building resilience against the challenges of raising a child with developmental differences,^57^ even in the context of social disadvantage.^63^

### Part C EI eligibility

Federal Part C legislation provides guidance but leaves it up to states to determine the specific criteria by which a child is eligible for EI services. Generally, eligibility for Part C EI is established by demonstrating that a child either has a developmental delay (delay-based eligibility, most common) or has been diagnosed with a condition with a high probability of resulting in developmental delay (diagnosis-based eligibility). Only six states (CA, FL, MA, NH, NM, WV) also serve infants and toddlers who are not yet showing delays but are considered “at risk” for reasons that can include exposure to psychosocial adversity and trauma.^54^

Diagnosis-based eligibility follows the logic that infants and toddlers already diagnosed with a condition known to result in developmental delays should be automatically eligible for Part C EI services. This pathway to eligibility is particularly relevant for infants diagnosed following NBSeq: having a genetic diagnosis at the point of entry to Part C EI could significantly increase a child’s chances of receiving services, regardless of whether they are yet showing delays that meet their states’ threshold for eligibility. Indeed, existing evidence suggests children who enter the system with a diagnosis (of any kind) are more likely to get, and potentially benefit from, Part C EI services.^64,65^ Presumptive eligibility via diagnosis-based eligibility could also be helpful for expediting the enrollment process, allowing for more efficient use of Part C EI program resources.^66^ However, the “Established Conditions” lists that are used to confer automatic eligibility differ widely across states. An analysis in 2019 found a total of 620 distinct conditions listed by at least one state, but no single condition was present on all lists, and 89% of conditions were listed by fewer than ten states.^67^ The three most commonly listed conditions were hearing impairment (38 states), fetal alcohol syndrome (34 states), and Down syndrome (32 states).

Therefore, whether a child will qualify for Part C EI services based on determination of developmental delay or diagnosis-based eligibility can differ greatly based on the state in which they live. States with broad eligibility criteria have greater participation in EI programs compared to states with narrow eligibility,^55,64,68^ and this difference particularly impacts poor children: the probability that a poor child (<100% federal poverty level) receives Part C EI is 18% lower if they live in a state with narrow vs. broad EI eligibility.^64^ Evidence suggests that states have attempted to reduce Part C EI expenditures by narrowing eligibility criteria,^55,68^ and the pressure to do so may be increasing as the amount of federal funding provided to states on a per-child basis has decreased 24% from $1616 in 2004 to $1222 per child in 2022 (unadjusted for inflation).^46^

### Lack of coordination between state NBS and Part C EI programs

In theory, infants diagnosed with a genetic condition that has a high likelihood of resulting in developmental delay should automatically qualify for EI services via diagnosis-based eligibility. But does this happen in practice? This is difficult to answer, as there is no national database that tracks children enrolled in Part C EI. However, insights can be gleaned from examining the extent to which children diagnosed with genetic disorders following traditional state-based NBS are granted automatic eligibility to Part C EI services.

Despite sharing similar goals—to ensure treatments or services for children begin as early as possible^56^—state-run NBS and Part C EI programs do not overlap administratively and, in most states, do not actively collaborate. A survey of state Part C EI and NBS coordinators found that the two groups had limited interaction and few coordinated processes for infants to enter Part C EI after an NBS diagnosis.^69^ Perhaps the most obvious coordinated effort would be to ensure that the conditions listed on a state’s NBS panel are also included on the state’s Part C EI established conditions list. However, currently, only one state (MI) automatically qualifies any child diagnosed through NBS for Part C EI. Most states include only a few NBS conditions (avg. 7.8) on Part C EI Established Conditions lists, and six states had no Established Conditions list at all. The divide between NBS and Part C EI condition lists is striking, given that an estimated 29/34 (85%) of RUSP conditions are associated with developmental delays and should thus automatically qualify a child for Part C EI.^14^ If more states follow the path of including genomic sequencing in NBS, the disconnect between the number of conditions included on NBS panels and those that confer automatic eligibility for EI will (in the absence of policy reform) only grow.

Aside from administrative/bureaucratic barriers, another potential reason for lack of collaboration between state-run NBS and EI programs is that the cultures of the two programs differ considerably.^56^ As a public health program, NBS is focused on preventing potentially devastating harm to affected infants.^17^ NBS programs might ask the question: “Is there net benefit to screening for condition X?”^70^ Part C EI, in contrast, has roots in special education and aims to improve outcomes for infants and toddlers by providing family-centered support. EI programs might ask the question “What does this family need to be able to support this child’s development?”^57^ Given that the majority of children gain access to Part C EI through delay-based eligibility, the services provided tend to be agnostic to diagnosis, focusing on what a child’s current needs are, rather than trying to identify the underlying reason for them. Therefore, the way that NBS programs, and most medical providers, might approach a case (diagnose, then treat) is different from the approach taken by Part C EI providers (evaluate, then support). In addition, the types of developmental supports offered in EI are not considered “treatment” and thus not included in net benefit calculations by NBS programs.^56^ The extent to which these divergent philosophies represent a true barrier to collaboration between public health NBS, medical systems, and EI is unknown and could be informed by comparison to the degree of collaboration seen in other countries.

## Questions to guide future research

Thus far we have established that through a combination of public health, clinical, and research efforts, the number of infants and toddlers with a genetic diagnosis made via newborn sequencing is expected to increase. This increase in the number of children with an identified need for developmental supports is likely to place a burden on the already overtaxed Part C EI system in the USA. Currently, EI does not adequately serve children who live below the poverty level or are from culturally or linguistically diverse backgrounds,^64,71,72^ and does not sufficiently coordinate with state-run NBS programs.^14,69^

Solving this challenge will likely require a multi-pronged approach including increased federal and state funding of Part C EI programs and system-wide policy change. For example, a national-level effort similar to the RUSP could harmonize Established Conditions lists for Part C EI programs and align this list with the RUSP, thus ensuring that all US children identified via NBS are able to access Part C EI services. Exactly which policy solutions are needed, however, requires further scholarship. In this final section and in Fig. 2, we pose research questions relevant to public health NBS, clinical care, NBSeq research, and EI that should be addressed to guide future policy initiatives.

As a first step, it is important to gain a better understanding of the developmental services landscape for infants with genetic diagnoses. Are parents able to leverage genetic diagnoses into developmental services, particularly within the Part C EI system? Do parents who receive Part C EI services feel that their child is appropriately supported by these services, and if not, why not? Infants with many genetic diagnoses face significant medical complexity, and parents take on the role of coordinating care across multiple specialists. For children served in the Part C EI system, how do EI providers and medical subspecialists interact to support a child’s care? How can this communication be improved? As described, most children served in Part C EI qualify on the basis of developmental delay, and the services provided are intended to support a child’s currently evident delays; services are not tailored to preemptively address potential delays associated with specific diagnoses. It is important, then, to understand how current EI providers working with children who qualify under diagnosis-based eligibility tend to incorporate that diagnosis into their treatment planning, and how a given diagnosis (particularly of a very rare condition) shapes the provision of Part C EI services, if at all. Finally, and critically, studies evaluating whether and how Part C EI services improve developmental outcomes for children with genetic diagnoses are lacking, and these studies will be critical for advocating for and designing system change.

Answering these initial empirical questions can set the stage for considering broader normative, systems-level questions in anticipation of broader implementation of NBSeq. How should Part C EI systems be structured to support needs of children with genetic diagnoses? As described, Part C EI is a limited resource, and already does not serve all children in need of developmental services. Is serving pre-symptomatic infants and toddlers with a genetic diagnosis, who are at risk for delays that in some cases may be mild and in others quite profound, the best use of Part C EI resources?^7^ How can the Part C EI system be leveraged to generate much-needed data on both the natural history/developmental functioning of young children with rare conditions as well as the efficacy of EI for improving developmental trajectories? When conducting research on NBS in the setting of a country like the USA with unequal access to Part C EI services based on geography and family circumstances, what are the “post-trial” responsibilities of researchers and funders to support families who learn of a genetic disorder via NBSeq?^73,74^

## Conclusion

Two decades after the completion of the Human Genome Project, technological innovations in next-generation sequencing have made it possible to screen every newborn for thousands of rare genetic conditions. However, normative questions about whether we should employ this technology remain; many of these focus on treatment availability and equitable provision of services. In the USA, additional work is needed to understand the strengths, limitations, and readiness of Part C EI—the primary system that supports the development needs of infants and toddlers—for serving the anticipated growing number of children with genetic diagnoses following newborn sequencing.
